# Trioecy is maintained as a time-stable mating system in the pink sea urchin *Toxopneustes roseus* from the Mexican Pacific

**DOI:** 10.1038/s41598-022-26059-4

**Published:** 2022-12-10

**Authors:** Julia Patricia Díaz-Martínez, Leobarda Margarita Mejía-Gutiérrez, Valentina Islas-Villanueva, Francisco Benítez-Villalobos

**Affiliations:** 1 Programa de Posgrado en Ecología Marina, División de Estudios de Posgrado, Universidad del Mar Campus Puerto Ángel, Cd. Universitaria S/N, 70902 Oaxaca, Mexico; 2 Consejo Nacional de Ciencia y Tecnología (CONACYT), Av. de los Insurgentes Sur 1582, 03940 Mexico, Mexico; 3 Instituto de Genética, Universidad del Mar Campus Puerto Ángel, Cd. Universitaria S/N, 70902 Oaxaca, Mexico; 4 Instituto de Recursos, Universidad del Mar Campus Puerto Ángel, Cd. Universitaria S/N, 70902 Oaxaca, Mexico

**Keywords:** Ecology, Evolution

## Abstract

Trioecy is a sexual system that consists of the co-occurrence of females, males and hermaphrodites in a population and is common in plants; however, in animals it is uncommon and poorly understood. In echinoderms, trioecy had never been recorded until now. Frequencies of females, males, and hermaphrodites were evaluated and gametogenic development was histologically characterized in a population of *Toxopneustes roseus* inhabiting the Mexican Pacific. Trioecy in this population is functional and temporally stable, since the three sexes coexisted in each sampling month. The hermaphrodites presented similar gametogenic development as the females and males and participated during the spawning season, contributing to the population’s reproductive process. Trioecy is considered an evolutionarily transitory state, and it is extremely difficult to explain its presence in a species. We hypothesize that continuous ocean warming represents a threat to the survival of this population of *T. roseus*, since its early developmental stages, which represent a population bottleneck, are more vulnerable to high temperatures than other sea urchins inhabiting the area, while its population density is significantly lower. These conditions generate a strongly stressed environment, which is the determining factor that maintains the stability of trioecy in the species in which it has been studied.

## Introduction

Reproduction and the way in which any living organism reproduces is the fundamental objective in its life history and important to ensure its permanence in ecosystems. Therefore, the biological success of any species involves its members remaining alive long enough to be able to reproduce^1,2^. In nature, there are two types of reproduction: sexual and asexual. Sexual reproduction consists of a complex and sophisticated mechanism, which involves the recombination of an individual´s own hereditary material and the exchange or fusion with the hereditary material of another individual. This type of reproduction increases genetic variability in offspring by producing unique combinations of genes inherited by parents, which makes possible the continuous biological evolution of living beings and their adaptation to the environment^3^.

Whereas different breeding systems exist amongst animals^4^, there are two common sexual systems, gonochorism (or dioecy), in which individuals are either female or male during their reproductive lives but not both, and hermaphroditism, in which the individual produces both male and female gametes during their breeding lives^5^. Most animal species are gonochoric with hermaphroditism occurring in a very low proportion (5–6%) of species^6^. In addition, there are rare mixed reproductive systems, including androdioecy (males and hermaphrodites), gynodioecy (females and hermaphrodites), and trioecy (co-occurrence of males, females, and hermaphrodites).

The condition of the coexistence of males, females, and hermaphrodites is a rare mating system that had previously been documented only in plants, and according to the theory of sex assignment, it should not occur in metazoans within discrete generations and considering simple theory^7^. However, some studies have recently shown that there is evidence of trioecy in two species of microscopic nematodes of the new genus *Auanema* (*A. rhodensis*, aka *Rhabditis* sp. SB347 and *A. freiburgensis*, aka *Rhabditis* sp. SB372), and three species of the genus *Tokorhabditis*^8–13^. This condition has also been recorded in the green hydra *Hydra* viridissima, the marine anemone *Aiptasia* diaphana, and the marine bivalve mollusk *Semimytilus algosus*^14–16^.

According to studies performed with plant populations, several different factors have been identified or proposed as important to promote the existence of different mating systems, sometimes within populations of the same species in close geographic proximity. For example, in the Sonoran Desert columnar cactus (*Pachycereus pringlei*), trioecy occurs near the known maternity roosts of its main pollinator, the nectarivorous bat *Leptonycteris curasoae*; while gynodioecy occurs > 50 km from bat roosts^17^. The observed geographic patterns cannot be explained by limited gene flow or geographic distributions of diurnal pollinators. Instead, it has been proposed that the abundance of chiropteran pollinators plays an important role in the maintenance of trioecy in this species, and under the limitation of pollinators, trioecy can be a stable reproduction system in this type of population^18,19^. Trioecy has also been evidenced in the knotweed *Coccoloba cereifera*, an endemic and endangered species from the rupestrian fields of Serra do Cipó, southeastern Brazil^20^. The flowers of this plant, like those of dioecious species, are small, unspecialized, and can attract several small insects. However, the limited number of visits to *C. cereifera* flowers is related to its presence at high altitudes, where potential pollinating invertebrates are restricted to anthophilous animals (commonly known as bees), especially social insects. Consequently, trioecy remains stable due to this limitation of pollinators^18–20^.

Another aspect that has been considered important is the low population density, as proved by the examination of the frequencies of females, males, and hermaphrodites across ten natural populations of the euphorbia *Mercurialis annua* and the evaluation of the association between the frequency of females and plant densities^21^. It was evident in that species that he stable co-existence of all three sex phenotypes within populations where likely only under low densities^21^. Finally, in studies with clones transplanted from the natural habitat to controlled conditions, variation occurred within irrigation treatments, between treatments, and over time, as a consequence of the combined effects of genotype plus environment in *Atriplex canescens* (Family Amaranthaceae)^22^. The magnitude of sex change was a product of the interaction of genetics and environment. Likewise, in an investigation of the breeding system of the ash tree *Fraxinus excelsior,* using field data and those from a seed orchard, it was found that trioecy is functional and the reproductive system of *F. excelsior* is not labile, as sex expression seems to be stable through time^23^. Finally, sex is genetically determined in this plant since different trees belonging to the same clone in the orchard exhibited similar sexual phenotypes.

It is known that taxa with well-developed mate search abilities tend to have separate sexes, while sessile taxa and those species with low mate search abilities tend to exhibit increased hermaphroditism^24^. Therefore, sessile organisms such as plants on land and many invertebrates in the marine environment are expected to exhibit higher levels of hermaphroditism than mobile organisms. It is also considered that different reproductive strategies have appeared during the evolutionary transition between dioecy and hermaphroditism or vice versa^4,25^. This type of transition has been better understood in plants than in animals and unlike plants, transitions in animals are mainly from dioecious to hermaphroditic systems^4,24,26–28^. The limited knowledge of this transition in animals is probably due to the lack of appropriate models that allow the establishment of plausible hypotheses. For that reason, it is essential to study species in which hermaphroditic and unisex animals coexist in the same discrete population to understand both the evolution of sexual systems and the causes that induce transitions.

In the phylum Echinodermata, dioecy is the most frequent reproductive strategy, and hermaphroditism is very rare and almost absent in echinoids^29–32^. Despite the low incidence of the finding in echinoids, hermaphroditism has been reported in some genera, such as *Arbacia*, *Paracentrotus* and *Strongylocentrotus*, and less frequently in *Echinus* and *Sphaerechinus*^33^. There was a record of a hermaphroditic individual in 3000 examined specimens of *Echinus esculentus*^34^. Also, a hermaphrodite was reported in 2350 analyzed specimens of *Arbacia punctulata*^35^. Likewise, there is a record of hermaphroditism in the genus *Tripnuestes*, which belongs to the same family as *T. roseus*^36^. In a recent case, only one case of ovotestis was recorded in *Loxechinus albus*, in a sample of 950 specimens. However, this could not be defined as hermaphrodite because there could be confusion between the process of early sexual differentiation that occurs during the juvenile or subadult stage and a clear "hermaphroditic" condition of an individual^37^.

*Toxopneustes roseus* is the species with the largest latitudinal distribution of the genus *Toxopneustes*, and it exhibits high ecological relevance due to its feeding habits and behavior. It plays a very important role in the contribution of carbonates to the water column, since it feeds mainly on crusty and calcareous algae unlike its congeners, making it distinctive as an important promoter of bioturbation. Very little is known about its reproduction although the reproductive patterns of other species of echinoderms have been characterized in the study area, which show a similar behavior in their gametogenic development and reproductive intensity throughout the year; however, none of these species has shown signs of hermaphroditism and even less of trioecy. The objective of this work is to describe in detail with histological analysis the condition of trioecy in adult specimens of *T. roseus* and to test the stability, temporal development (over a year), and functionality of this condition in a population that inhabits the Pacific region of southern Mexico.

## Material and methods

### Field work

**Figure 1:**
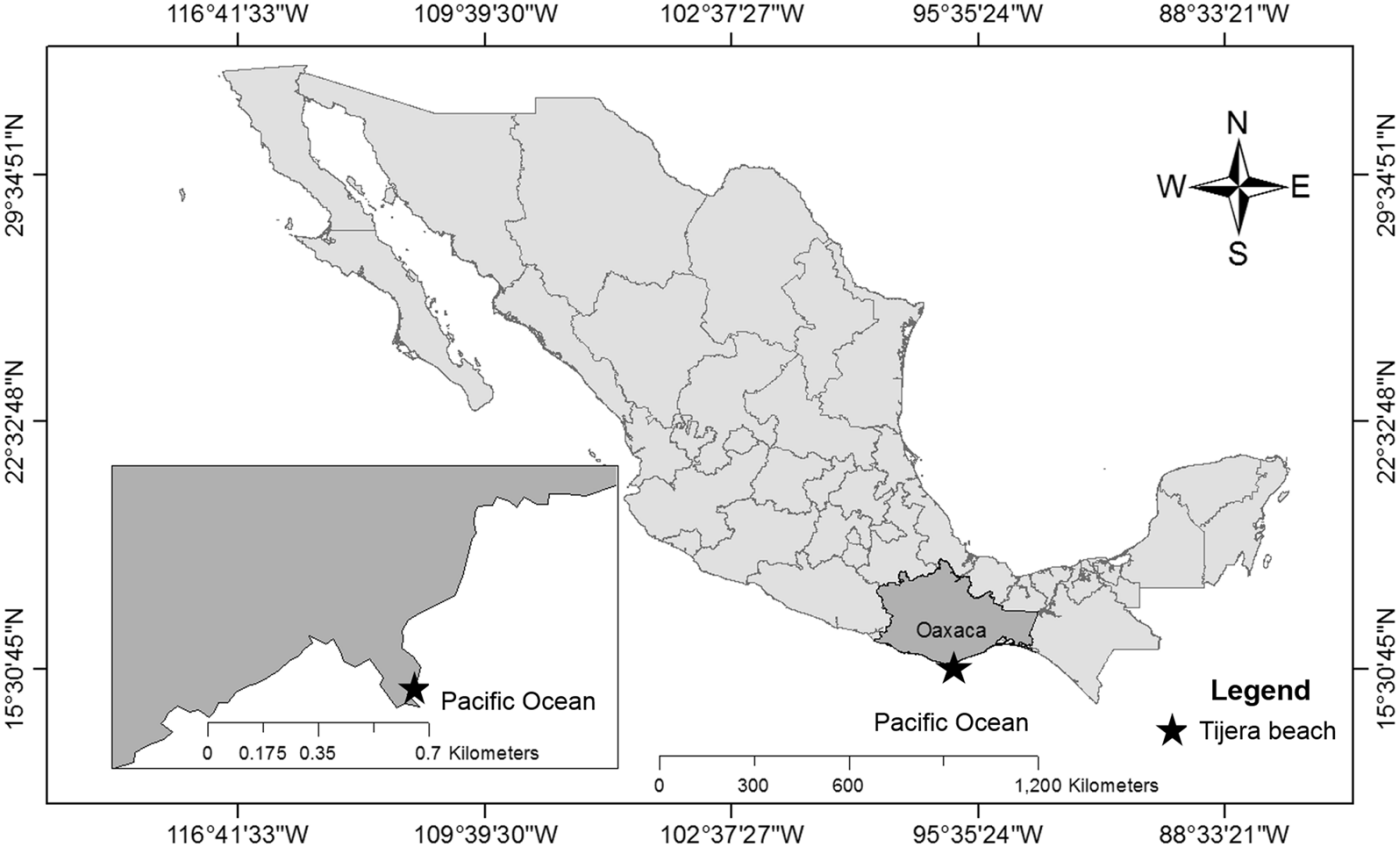
Location of the study area showing the position of Playa Tijera. The map in this figure was created with ArcMap Desktop (Version 10.8), https://desktop.arcgis.com/es/arcmap.

Between 18 and 21 specimens (except in March 2015) of *T. roseus* were randomly collected every month through SCUBA diving from March 2015 to April 2016 in Playa Tijera, Oaxaca, Mexico (15° 43ʹ 08.55ʺ N, 96 09ʹ 44.74ʺ W), which is a semi-protected bay surrounded by cliffs. The bay has a thick sandy substrate, a moderate slope and a depth of approximately 12 m (Fig. 1). The bottom has uniform extensions of coral reefs parallel to the coastline and sandy extensions with fragments of dead coral.

All the sea urchins collected (n = 224) had more than 50 mm of test diameter; therefore, they were adults that had already reached the size of sexual maturity^38^. The specimens were transported to the Laboratory of Development Ecology (ECODES) at the Universidad del Mar in Puerto Ángel, Oaxaca.

### Laboratory work

In the laboratory, the specimens were left in sea water at 4 °C for 24 h to sacrifice them without causing any suffering. After that lapse, they were dissected to obtain the gonads and the diameter of every individual was measured. The gonads of every specimen were fixed in Bouin's solution for 72 h, washed with tap water and preserved in 70% ethanol. Samples of each gonad were dehydrated in a series of graded ethanol solutions (1 h baths with 2 changes for each solution of 80, 90, and 100% ethanol), cleared in Citrisolv® (60 min baths with 2 changes), infiltrated in Paraplast paraffin with a 56 °C melting point (90 min baths with 2 changes) and embedded in Paraplast paraffin with the aid of a Histokinette. Serial sections were cut between 5 and 8 μm thickness using a manual rotary microtome (LEICA RM2145), later mounted on glass slides, warmed for 24 h at 50 °C in a furnace, and immediately stained with the routine Mayer hematoxylin–eosin progressive method. Digital images were taken with a camera (Karl Zeiss AxioCam c5S) mounted on a microscope (Karl Zeiss PRIMO STAR) and stored in a computer in graphic format.

Based on the analysis of the histological sections, the sex of each specimen was determined as well as the reproductive stage in which it was found. The followed criteria consisted of the distinctive cellular characteristics of the nucleus, cytoplasm, and follicular and germinal epithelium walls, as well as the distribution and size of the gametes in the gonadal follicles^39,40^. For the specific case of oocytes, the nucleus-cytoplasm relationship was also considered, as well as their affinity for the dyes.

Welfare, humanitarian sacrifice, and ethically responsible research with the sea urchins were in accordance with the ethical recommendations for humanitarian killing of animals as established under Mexican law (NOM-033-SAG/ZOO-2014). No live vertebrates were handled during this study.

### Data analysis

The sexual proportion of the specimens was calculated considering males, females and hermaphrodites. The proportions were analyzed using the Chi square test (*X*^2^) to determine if there was a significant deviation from a 1F: 1 M: 1Hp (Female: Male: Hermaphrodite) ratio. The decision rule was made with a 95% confidence interval, rejecting a 1: 1: 1 ratio when the calculated *X*^2^ value was greater than 5.99^41,42^.

## Results

**Figure 2:**
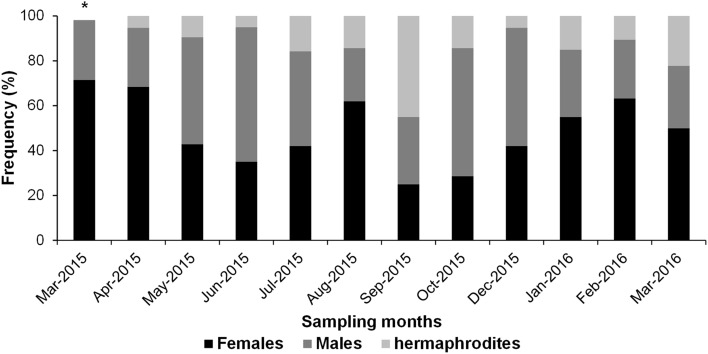
Frequency of sexes (%) of *T. roseus* during the sampling period. The asterisk indicates the month in which hermaphroditic individuals were not found.

Of the 224 specimens analyzed, there were 106 females (47.32%), 86 males (38.39%), and 32 hermaphrodites (14.28%) showing an annual sexual ratio (F: M: Hp) of 1.2: 0.8: 0.3, which differs significantly from a 1: 1: 1 ratio (X^2^ > 5.99, df = 11, α = 0.05) (Table 1). Individuals that presented hermaphroditism were observed in 11 of 12 months sampled, although the absence of hermaphrodites in March 2015 was probably due to the small sample size (n = 7). The highest incidence was observed with eight specimens (28.57%) in September 2015, while in the rest of the months the proportion was relatively low (3.1–12.1%) (Fig. 2).

**Table 1:** Monthly sex ratio for *Toxopneustes roseus* at Playa Tijera Oaxaca.

Months	M	F	Hp	n	*X*^2^	M:F:Hp
Mar-2015	2	5	0	7	18.4	1:2.5:0
Apr-2015	5	13	1	19	62.0	1:2.6:0.07
May-2015	10	9	2	21	25.9	1:0.9:0.2
Jun-2015	12	7	1	20	45.5	1:0.6:0.08
Jul-2015	8	8	3	19	13.9	1:1:0.37
Aug-2015	5	13	3	21	38.1	1:2.6:0.6
Sep-2015	6	5	9	20	6.5	1:0.8:1.5
Oct-2015	12	6	3	21	28.6	1:0.5:0.25
Dec-2015	10	8	1	19	37.1	1:0.8:0.1
Jan-2016	6	11	3	20	24.5	1:1.8:0.5
Feb-2016	5	12	2	19	43.8	1:2.4:0.4
Mar-2016	5	9	4	18	13.0	1:1.8:0.8
Total	86	106	32	224	357.2	0.8:1.2:0.3

*M* males, *F* females, *Hp* hermaphrodites, *n* total individuals analyzed per month.

**Figure 3:**
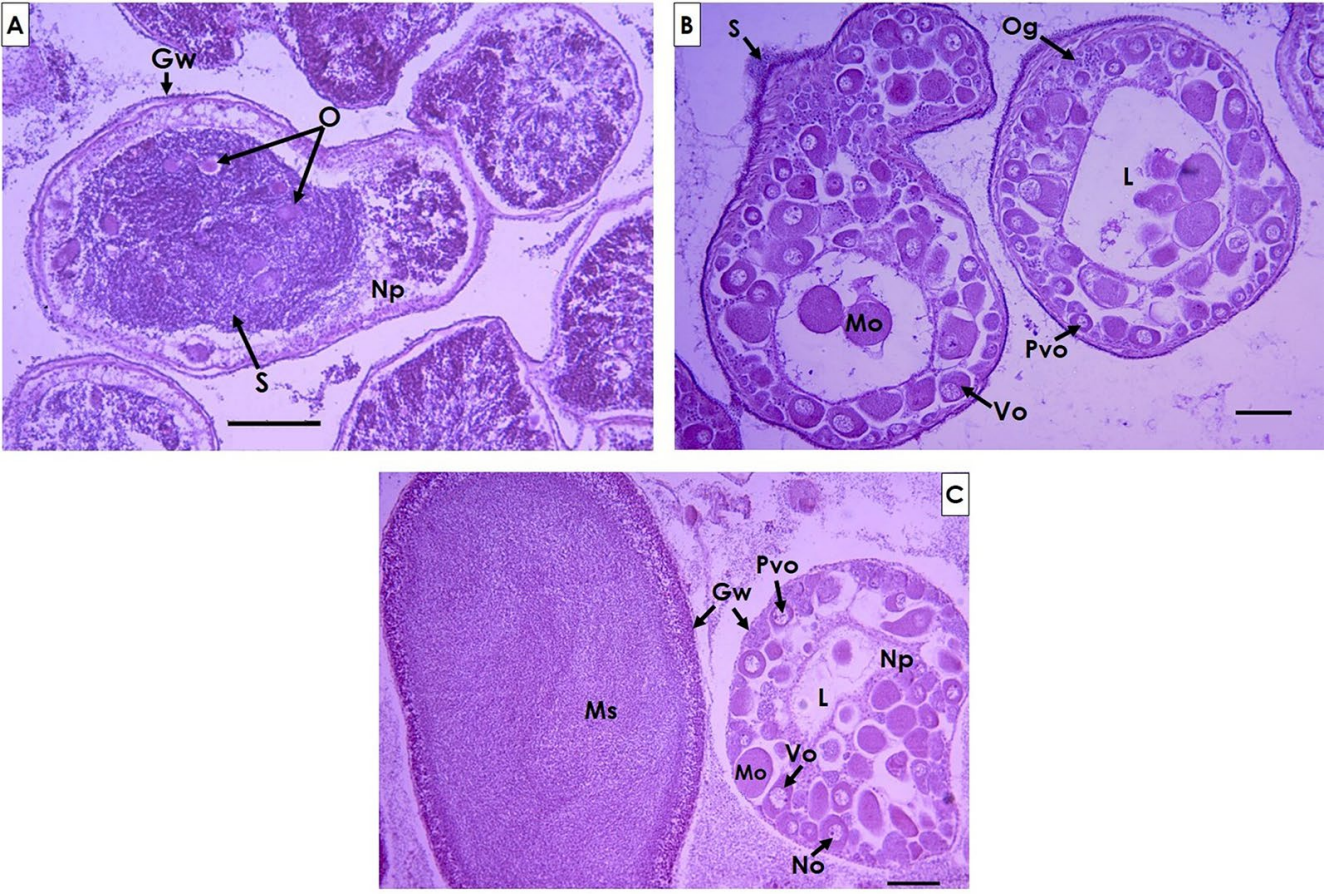
Gonad section of specimens with the presence of hermaphroditism, (**A**) gonad section with a larger presence of male gametes, (**B**) gonad section with a larger presence of oocytes, and (**C**) gonad with a similar proportion of both gametes. *Gw* gonad wall, *L* lumen, *Mo* mature oocytes, *Ms* mature spermatozoa, *No* nucleolus, *Np* nutritive phagocytes, *O* oocytes, *Og* oogonia, *Pvo* previtellogenic oocytes, *S* spermatids, *Vo* vitellogenic oocytes. Scale bars are 100 µm.

Of the 32 specimens with the presence of gametes of both sexes inside or outside the follicles, 27 (84.3%) had a higher proportion of male gonadal tissue (Fig. 3A), while three of the individuals (9.3%) had a higher presence of female gonadal tissue (Fig. 3B). Only two individuals (6.2%) presented the same proportion of tissue of both sexes (Fig. 3C).

**Figure 4:**
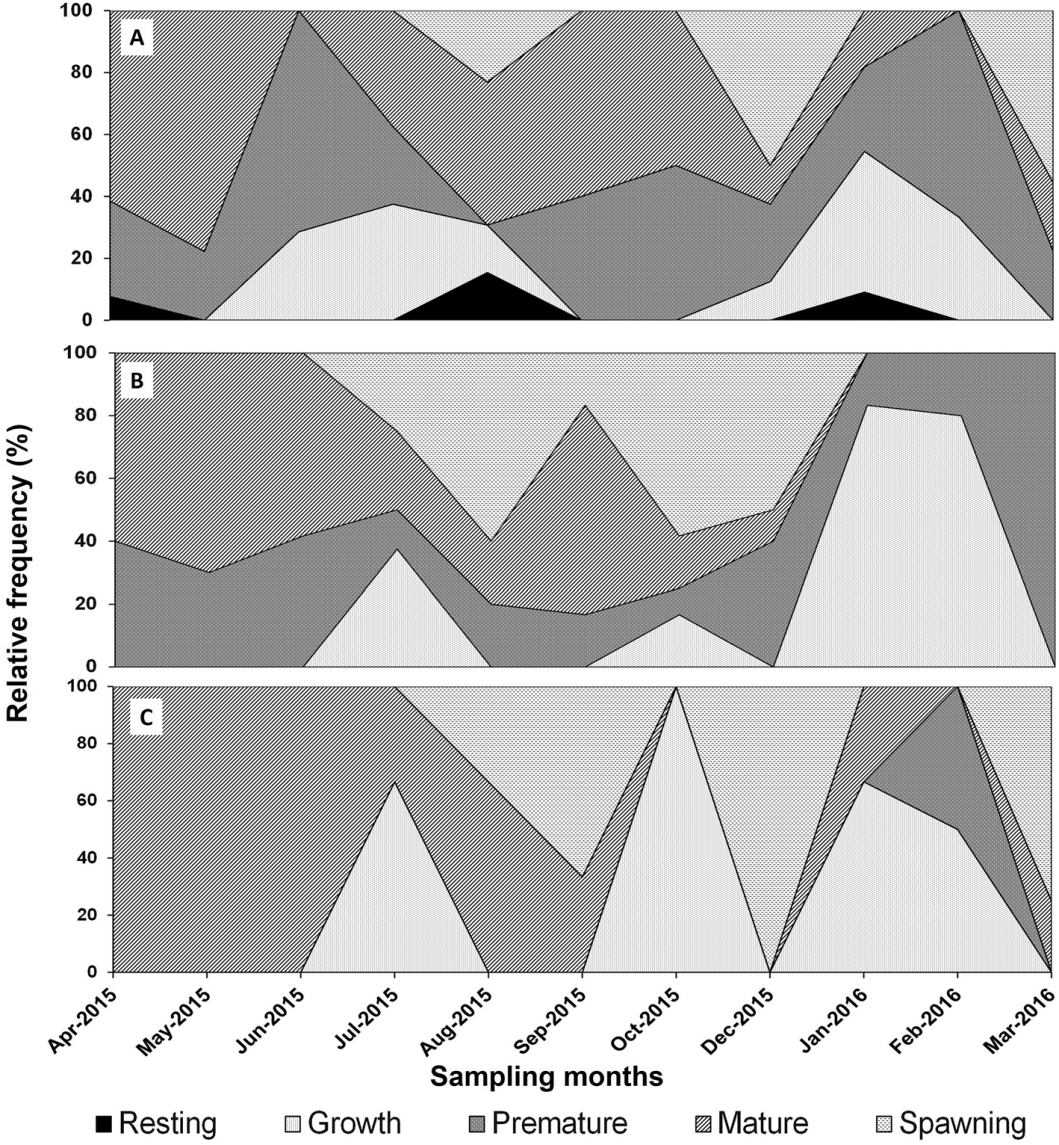
Relative frequencies of the reproductive stages of *Topxopneustes roseus* throughout the sampling period, (**A**) females, (**B**) males, and (**C**) hermaphrodites.

When analyzing the phases of gonadal development by sex, in females the resting phase occurred in three months (April 2015, August 2015, and January 2016), and the highest frequency of individuals in this phase occurred in August. The growth phase occurred almost every month, except in April, May, September, and October 2015. The prematurity phase occurred throughout the study period, except in August. The months with the highest proportion of maturity were March, May, and September 2015. The months in which no maturity was recorded were June 2015 and February 2016. Spawning events were recorded in August and December 2015, as well as in March 2016 (Fig. 4A).

In males, the resting phase did not appear. The growth phase was observed over four months, with the highest frequency in January 2016. The prematurity phase occurred synchronously with that of the females. The maturity phase began in April and ended in December 2015, and the highest frequencies of individuals in this stage were observed in May and September. Spawning occurred from July to December with the highest frequencies in August and October 2015 (Fig. 4B).

In hermaphrodites, the resting phase did not appear. The growth phase was observed in four months. The prematurity phase was only observed in February 2016 with a frequency of 50%. Maturity was recorded in the first three months with a frequency of 100%. The individuals began to spawn in August, increased in September, and all were spawning in December 2015 and again in March 2016 (Fig. 4C).

Histologically, the gonadal development stages of hermaphrodites were similar to those of dioecious specimens. The ovarian proportions possessed all cell types showing normal progression of oocyte formation from oogonia.

**Figure 5:**
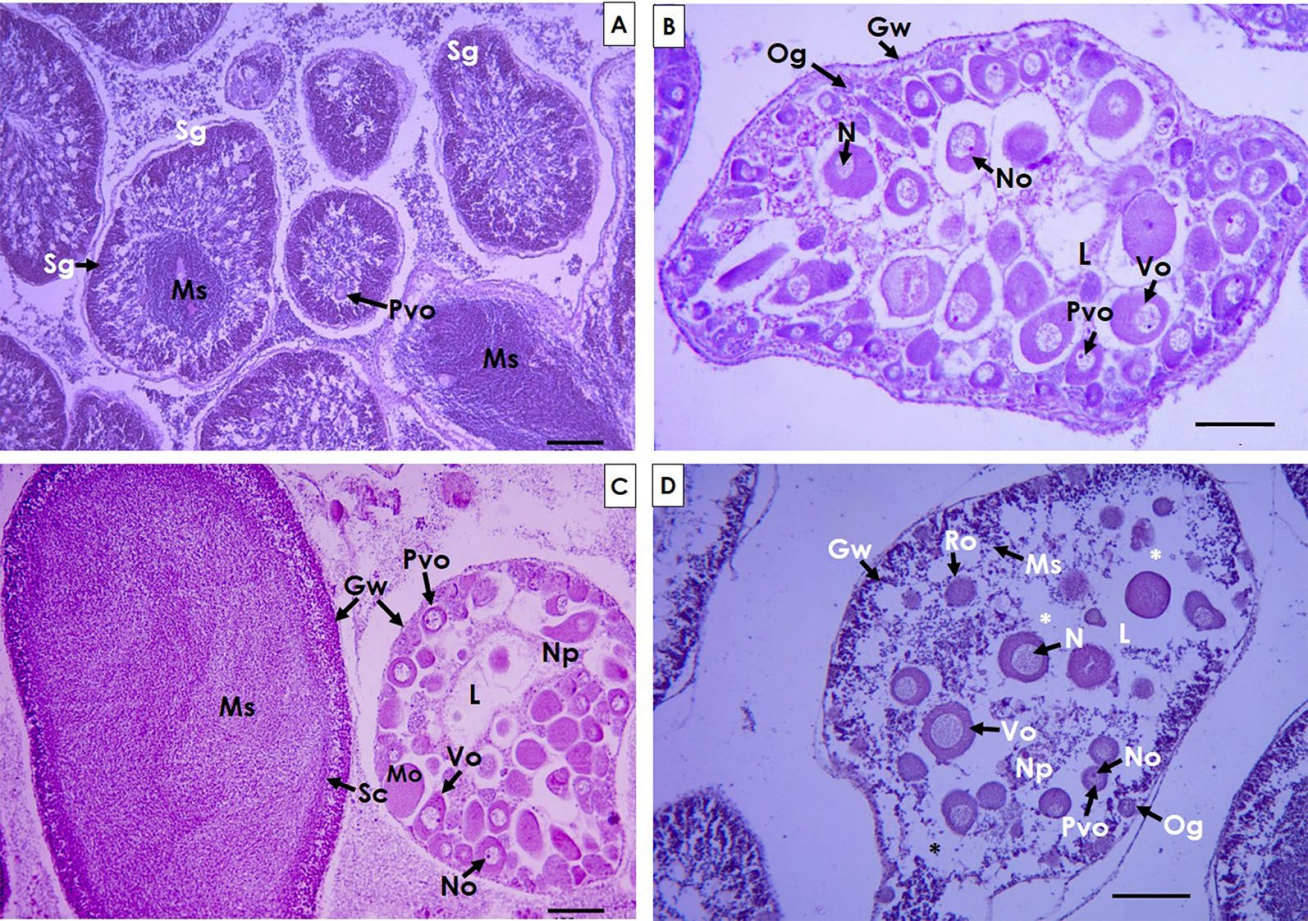
Stages of gametogenic development in hermaphrodites of *T. roseus*: (**A**,**B**) growth stage, (**C**) maturation stage, (**D**) spawning stage. *Gw* gonad wall, *L* lumen, *Mo* mature oocytes, *Ms* mature spermatozoa, *N* nucleus, *No* nucleolus, *Np* nutritive phagocytes, *Og* oogonia, *Pvo* previtellogenic oocytes, *Ro* remaining oocytes, *Sc* spermatogenic columns, *Sg* Spermatogonia, *Vo* vitellogenic oocytes. The asterisk indicates empty spaces due to the release of gametes. Scale bars are 100 µm.

In the growth stage, the presence of spermatogonia was observed in the periphery of the follicular wall in the gonads that contained developing male gametes. This is where spermatogenesis began heading towards the center, which concentrated the spermatozoa in the lumen of the follicle. The gametes were dark purple in color. In some follicles the presence of previthellogenic oocytes was observed, of which some were located near the periphery of the wall and others in the center of the lumen (Fig. 5A). In follicles containing female gametes, the presence of oocytes at different stages of development was observed. Previtellogenic and vitellogenic oocytes as well as nutritive phagocytes were observed in the lumen. The gonadal wall was thick, and previtellogenic oocytes and oogonia were observed near the periphery of the follicle (Fig. 5B).

In maturing hermaphrodites, follicles that only contained sperm could be identified, while in others there were only oocytes. The follicles containing only spermatozoa were characterized by presenting mature spermatozoa in the lumen and a band of primary sex cells was observed near the wall of the follicle (Fig. 5C). In the case of follicles with oocytes, these were observed at different stages of development, from oogonia to mature oocytes. The presence of a well-defined nucleolus was observed in previtellogenic oocytes, and most of these were found in the periphery of the follicle (Fig. 5C).

The individuals that presented follicles that were releasing spermatozoa were characterized by presenting empty areas between spermatozoa, and in some cases in which the release was partial, the gonadal wall was thin. Likewise, in some follicles the release of oocytes could be observed, which was characterized by presenting spaces that had previously been occupied by mature oocytes in the lumen, and the presence of remaining oocytes was also recorded (Fig. 5D).

## Discussion

According to the information that exists so far regarding reproduction in echinoderms, this is the first work in which the occurrence of trioecy in sea urchins is reported. This is also the first report of trioecy among members of the phylum Echinodermata, one of the most widespread taxa, both latitudinally and bathymetrically. Our results show that trioecy in this population of *T. roseus* is temporally stable, since the three sexes were observed together throughout the year in each month of sampling. Hermaphroditic individuals also presented the same gametogenic developmental pattern as females and males. Finally, during the spawning period of the population they contributed to the reproductive process by releasing mature gametes, which evidenced their full functionality within the studied population.

We were unable to obtain evidence of self-fertilization in the studied hermaphrodites; but self- fertilization in the gonads and gonadal ducts of a hermaphrodite individual of *Echinocardium cordatum* was recorded in 1935^43^. However, the embryos produced did not complete development successfully, probably due to the premature fertilization within the gonad^43^. Also, the cases of fully functional hermaphrodites of *Arbacia punctulata* have been reported^44,45^. The gametes of the hermaphrodites were fertilized as soon as they were released into seawater and the development of self-fertilized eggs was absolutely normal in time and morphology. After nine days, typical pluteus larvae were obtained and both the eggs and sperm of the hermaphrodites functioned ordinarily with gametes from other males and females.

Therefore, we consider that there are no reasons to think that in the case of *Toxopneustes roseus* hermaphrodites cannot carry out self-fertilization. According to the analysis of the gonad developmental stages, their gametes were released into seawater. Theoretically, those gametes would be able to follow the normal course of fertilization, interacting among them and with gametes of females and males.

The trioecic condition has been recorded so far only in some animals, such as a few nematode species and a hydra^9,10,14,46–48^. In marine invertebrates, it has been reported in one anemone under laboratory conditions and in one bivalve mollusk^15,16^. The coexistence of males, females and hermaphrodites has been considered an evolutionarily transitory state; for example, androdioecy (male / hermaphrodite) in nematodes such as *Caenorhabditis elegans* is believed to have evolved from dioecy (male / female) through a trioic intermediate. Consequently, it is very difficult to find the ecological or evolutionary causes that lead a species or population to present three sexes simultaneously^49^.

In the species in which trioecy has been studied and monitored, it is noticeable that their populations are subjected to strong environmental stress in situ or under laboratory manipulation^50–52^. For example, some nematodes of the genus *Tokorhabditis* are extremophilic species that live in the Californian Mono Lake, which is characterized by being hypersaline and exhibiting high levels of arsenic^10,50^. In the case of *Auanema freiburgensis* the flexible sex determination and mating system and, consequently, its trioecy can be critical for resilience at the population level in patchy, resource-limited environments^49^. These results thus demonstrate that life-history, ecology and environment can play defining roles in the development of sexual systems and determine the continued presence of trioecy in the nematode. In the case of *Hydra viridissima*, it unlike most European species, is a "warm crisis" hydra, since it usually reproduces asexually, but when the temperatures rise to, or are maintained at high levels (≥ 20 °C), it reproduces sexually^14,53^. In experimental conditions, the population studied essentially behaved as androdioecic and only at the end of the research period, when the temperature was the highest (~ 25 °C), a few females appeared and joined the other existing sexes, thus generating the condition of trioecy^14^. Trioecy has been identified in another non-described species (e.g*., Rhabditis* sp. JU1783) isolated from star fruit, although it is closely related to *A. rhodensis* and *A. freiburgensis* and likely to belong to the same genus^11,12^. Little is known about the ecology of *Auanema*, as *A. rhodensis* has been isolated from a tick and a beetle, and *A. freiburgensis* from dung and a rotting plant of the genus *Petasites*^12,47,51^.

Regarding the sea anemone *Aiptasia diaphana*, it is mainly found in isolated fouling communities, and no hermaphrodites exist in natural populations that could reproduce asexually or sexually^54^. However, under laboratory conditions, a single founder individual (asexual clone) produced not only males and females, but also hermaphroditic individuals. In addition, *A. diaphana* can fertilize within and between cloning lines, producing larval-swimming planules, which could explain the success of the species as an invader of artificial marine substrates. The condition of trioecy was also identified in individuals of this anemone manipulated in the laboratory, to create age-homogeneous populations of asexual propagules (pedal lacerations) and ontogenetic patterns of sexual differentiation were documented^15^.

In the case of the marine bivalve *Semimytilus algosus*, there was not an obvious explanation for the occurrence of its trioecy, despite the intense analyses of factors such as motility versus a sessile way of life or reproductive density within a population, which could have relevance for gamete interactions^16^. In many respects, *S. algosus* is a “typical” marine intertidal mussel, since it is sessile in adulthood, occurs at high densities in wild populations, and has a very large population. *S. algosus* also co-occurs with other species that are close relatives within the Mytilidae family and have evolved and conserved their dioecy^16^.

*Toxopneustes roseus* is another typical species of sea urchin, which has a wide latitudinal distribution throughout the tropical eastern Pacific and co-inhabits with other species of sea urchins and echinoderms that have a similar distribution and in which hermaphroditism has not been reported^40,55–57^. Regarding its population density, T. *roseus* is not considered among the most abundant species in the study area and its densities are relatively low (between 0.04 and 1.2 ind.m^2^). However, it cannot be considered a rare species in terms of abundance^58,59^.

All of the above makes it difficult to clearly explain the reasons for the occurrence of trioecy in this species; however, certain aspects of its early development are known that could indicate the factors behind the development of this reproductive mating system in the pink sea urchin. In recent experiments carried out with gametes, larvae, and embryos of a population of *T. roseus* from the same area as our study, it was found that the increase in temperature above the normal values of its habitat has a deleterious effect on the success of early development^60^. There exists experimental evidence that at an increase of temperature to 32 °C, which is 2 °C above the maximum values registered in the study area, fertilization occurred at a very low percentage. There was also a deleterious effect on embryos, resulting in abnormal development and the lowest percentage of larval survival also occurred at 32 °C^60^. The same kind of experiments has been performed on other species from the study area, such as the irregular sea urchin *Ryncholampas pacificus* and the intertidal *Echinometra vanbrunti*. The deleterious effects on these species were observed only at 34 °C, which was the highest temperature tested (unpublished data). At 32 °C, however, there was no evidence of negative effects in the case on *E. vanbrunti*, and there was just arrested development, but no abnormalities in the case of *R. pacificus*. These results indicate that *T. roseus* is much more sensitive to the rise in temperature than other cohabiting sea urchins, and probably lives near its upper thermal limit. In that context, the continuous ocean warming could threaten the permanence of the species in the study area, since the early stages of development constitute a bottleneck for successful recruitment and later population maintenance in populations that carry out reproduction by means of external fertilization.

Within the phylum Echinodermata, when stressful conditions appear in the habitat or the environment becomes hostile, the species can generally resort to asexual reproduction by fission (ophiuroids) or fission and autotomy (holothuroids and asteroids) to increase the abundance of populations in a relatively short time or counteract a threat with numbers^61^. This does not apply to sea urchins since they are unable to reproduce asexually. The only way for sea urchins to reproduce asexually would be by cloning larvae, but this process would also require that sexual reproduction occurs first^62^. Therefore, any reproductive strategy that a sea urchin population could develop to respond to drastic changes in their area must involve sexual reproduction. In this regard, in an experimental evolution study with the nematode *Caenorhabditis elegans,* in which partial selfing, exclusive selfing, and predominant outcrossing were compared, it was evidenced that monoecious populations only have hermaphrodites and, therefore, reproduction is carried out exclusively by self-fertilization. However, in trioic populations that have males, females, and a small number of hermaphrodites, reproduction is predominantly carried out by external crossing^49^. Also populations that underwent some degree of interbreeding during the evolutionary experiments (trioic and androdioic populations), maintained more genetic diversity than expected solely under genetic drift or under genetic drift and directional selection^49^. In this sense, it is possible that high levels of interbreeding, such as that which occurs in trioic populations, develop with populations that have sufficient deleterious recessive alleles to avoid extinction, since selection is less efficient to purge them. Trioecy, therefore, becomes an efficient system to select characteristics of the genome that allows a population that only reproduces sexually to adequately cope with significant changes in the environment that could threaten the permanence of the species in that habitat. Interbreeding (gonochorism, self-incompatible hermaphroditism) also favors genetic diversity and offers greater potential to adapt to changing environments^63^. The costs and advantages of crossing over selfing depend on environmental factors and, therefore, selection may favor transitions between mating systems. Androdioecy, gynodioecy, and trioecy are evolutionarily unstable intermediate strategies, but they offer important systems for testing models of the causes and consequences of the mating system in the evolution of populations^63^.

However, the question remains why *T. roseus* has developed trioecy, when in the same habitat there are other sea urchins with very similar life-histories that only maintain dioecy. In the case of the bivalve *Semimytilus algosus*; which presents the same situation as we have with *T. roseus,* it was proposed that the trioecy of the species may be related to the sex determination mechanism, considering what it is known about the nematodes of the genus *Auanema*^10,16,46^. In *Auanema*, the male versus non-male (hermaphrodite or female) decision is determined genetically (XO for males, and XX for females and hermaphrodites)^9,64^. The hermaphrodite versus female decision, however, is determined by the environment of the mother. For *A. freiburgensis* the maternal social environment is determinant, whereas for *A. rhodensis* it is the age of the mother^9,12,51,65^. Therefore, in *Auanema,* environmental sex determination and genetic sex determination interact to produce trioecy.

Although there is apparently no clear cause of strong, stressful conditions in the habitat of *T. roseus* that could threaten the survival of this species, according to the United States Environmental Protection Agency (EPA, 2021), sea surface temperature increased during the twentieth century and continues to rise. From 1901 to 2020, the global temperature rose at an average rate of 0.004 °C per decade, resulting in a total increase of 0.5 °C to date. Additionally, regional studies based on continuous monitoring, which have not yet been published, have shown that between 2002 and 2020 there has been an increase of approximately 1 °C above the historical average of the sea surface temperature in the study area.

The foregoing discussion leads us to speculate that the studied population of *T. roseus* lives at the limit of its thermal tolerance, and the constant increase in ocean temperature due to global warming constitutes a threat to its survival and a constant source of stress for the population. This is because its early-development stages are more vulnerable to high temperature than other sea urchins that live in the same area and its population density is also significantly lower^58^.

Phylogenetically *T. roseus* belongs to Family Toxopneustidae and although no other species within the genus *Toxopneustes* has shown hermaphroditism, this condition was reported in *Tripneustes gratilla*, which belongs to the same family^36^. Toxopneustids belong to the Order Camarodonta, and almost all the species of sea urchins in which hermaphroditism has been reported belong to this Order except for a couple that belong to the Arbacioida. At the same time, this order is contained in the Superorder Echinacea along with Camarodonta, according to the last exhaustive analysis resolving the position of the clades within Echinoidea^66^. In this context, theoretically *T. roseus* at some point underwent the environmental pressure of its early stage living under constantly rising temperatures, along with its low population densities in the study area. Consequently, it was able to develop hermaphroditism and, therefore, trioecy, similarly to what occurred to *Hydra viridissima* under conditions of extreme high temperature^14^. We hypothesize that these permanent conditions generate a constant source of strong environmental stress, which is the determining factor that keeps trioecy stable in the species in which it has been studied, and, thus, trioecy remains stable in this population of *T. roseus*.

The mechanism of sex determination in echinoids, as well as in other echinoderms, is still unknown, although the sex ratio, which is generally close to 1:1, suggests that it occurs through sex chromosomes^67^. It is known that in mammals, sex determination is dictated by the presence or absence of the Y-chromosomal gene SRY. SRY functions as the primary sex-determining gene by activating testis formation, and in its absence, the embryo will form ovaries. SRY only exists in mammals; however it evolved as a duplication of the *Sox* gene family, which exists in all metazoans^68^.

In vertebrates, *Sox* genes are involved in sex determination, neurogenesis, skeletonogenesis, eye development, pituitary development, pancreas formation, and neural crest and notochord formation^69^. In invertebrates, they are involved in processes such as metamorphosis, eye development, neural crest formation, and ectoderm formation^70^. In the sea urchin *Strongylocentrotus purpuratus*, *SoxB1* was determined to be expressed in the primordial gut during development and is closely related in sequence to *Sox* genes of the mouse embryo^71^. An investigation of sex determination was carried out in the sea urchin *Strongylocentrotus purpuratus* using RNA-seq and quantitative mRNA measurements, but the mechanisms that govern sexual determination of the species could not be clearly established^72^. However; the results show that the male fate factors *Dmrt* and *SoxH* are expressed early and meiosis initiates early. Also, gonad-specific transcripts involved in egg and sperm biology, are first activated before rudiment formation in the larvae of this sea urchin. The study provided additional evidence for the hypothesis that in sea urchins, sex determination occurs genetically^72^. Another research with the sea cucumber *Apostichopus japonicus*, which integrated genome-wide association study and analyzes of sex-specific variations evidenced that the species exhibits genetic sexual determination^73^. Furthermore, analysis of homozygous and heterozygous genotypes of abundant sex-specific SNPs in females and males, confirmed that *A.japonicus* might have a XX/XY sex determination system^73^.

On the other hand, it has been proposed that a deviation from the 1:1 sex ratio in echinoids could reflect environmental conditions that influence sex determination^67^. For example, a relatively large proportion of *Lytechinus variegatus* and *Tripneustes ventricosus* (as *Tripneustes esculentus*) hermaphrodites was recorded in southern Florida during an unusually cold winter, suggesting that adverse winter conditions in some way affected sex determination in juveniles^74,75^. Also relatively large number of *Strongylocentrotus purpuratus* hermaphrodites was reported in Bahía de Todos los Santos, Mexico, where extreme seasonal fluctuations in temperature (from about 12–24 °C) are recorded^76^. However, posterior studies did not find a single hermaphrodite of *Strongylocentrotus purpuratus* in more than 500 individuals analyzed^77,78^.

Considering that sex determination in sea urchins is highly probable to occur genetically and the possibility that the environment may also influence sex determination, we think that in the case of *Toxopneustes roseus*, genetic sex determination and environmental sex determination are interacting to maintain the condition of trioecy stable. We propose that, especially because the cases in which environmental conditions have assumed to influence sex determination, extreme temperatures are invoked as the main affecting factor. However, more detailed studies are needed in terms of sexual determination and experimental evolution to be able to verify our assumption.

In general, the efforts that have been made to explain the evolution of the sexes and the origin of hermaphroditism and trioecy are still scarce, and critical questions remain to be answered. The case of trioecy detected in *T. roseus* may constitute an important model to seek these answers about the evolution of sexual systems and the environmental mechanisms that trigger trioecy in marine macroinvertebrates and, in particular, in echinoderms.

## Data Availability

The datasets generated during and/or analyzed during the current study are available from the corresponding author on reasonable request.
